# Evaluation of click chemistry microarrays for immunosensing of alpha-fetoprotein (AFP)

**DOI:** 10.3762/bjnano.10.241

**Published:** 2019-12-16

**Authors:** Seyed Mohammad Mahdi Dadfar, Sylwia Sekula-Neuner, Vanessa Trouillet, Hui-Yu Liu, Ravi Kumar, Annie K Powell, Michael Hirtz

**Affiliations:** 1 Institute of Nanotechnology (INT), Karlsruhe Institute of Technology (KIT), Hermann-von-Helmholtz-Platz 1, 76344 Eggenstein Leopoldshafen, Germany; 2 Karlsruhe Nano Micro Facility (KNMF), Karlsruhe Institute of Technology (KIT), Hermann-von-Helmholtz-Platz 1, 76344 Eggenstein Leopoldshafen, Germany; 3 Institute for Applied Materials (IAM), Karlsruhe Institute of Technology (KIT), Hermann-von-Helmholtz-Platz 1, 76344 Eggenstein Leopoldshafen, Germany; 4 Institute of Inorganic Chemistry (AOC), Karlsruhe Institute of Technology (KIT), Engesserstraße 15, 76131 Karlsruhe, Germany

**Keywords:** alpha-fetoprotein (AFP), cancer biomarker, click chemistry, fluorescent immunosensor, hepatocellular carcinoma

## Abstract

The level of cancer biomarkers in cells, tissues or body fluids can be used for the prediction of the presence of cancer or can even indicate the stage of the disease. Alpha-fetoprotein (AFP) is the most commonly used biomarker for early screening and diagnosis of hepatocellular carcinoma (HCC). Here, a combination of three techniques (click chemistry, the biotin–streptavidin–biotin sandwich strategy and the use of antigen–antibody interactions) were combined to implement a sensitive fluorescent immunosensor for AFP detection. Three types of functionalized glasses (dibenzocyclooctyne- (DBCO-), thiol- and epoxy-terminated surfaces) were biotinylated by employing the respective adequate click chemistry counterparts (biotin–thiol or biotin–azide for the first class, biotin–maleimide or biotin–DBCO for the second class and biotin–amine or biotin–thiol for the third class). The anti-AFP antibody was immobilized on the surfaces via a biotin–streptavidin–biotin sandwich technique. To evaluate the sensing performance of the differently prepared surfaces, fluorescently labeled AFP was spotted onto them via microchannel cantilever spotting (µCS). Based on the fluorescence measurements, the optimal microarray design was found and its sensitivity was determined.

## Introduction

Hepatocellular carcinoma (HCC) is the major cause of morbidity and mortality in patients with chronic liver disease, the sixth most common cancer and the fourth leading cause of cancer-related deaths worldwide. The last release of the Global Cancer Observatory (GCO) database in September 2018 estimated about 840,000 new cases and 780,000 deaths of liver cancer in 2018 for both sexes and all ages [1]. Cirrhosis of the liver, hepatitis B virus (HBV) and hepatitis C virus (HCV) infections, heavy alcohol consumption, ingestion of aflatoxin and certain diseases like hemochromatosis, alpha 1-antitrypsin deficiency (A1AD or AATD) and nonalcoholic steatohepatitis (NASH) are the most important risk factors for HCC development [2–4].

The life expectancy of HCC patients depends on the stage of the disease at detection. A diagnosis of HCC at early stage through surveillance methods provides highly effective treatment and prolongs the lifetime of patients. However, when the disease is detected in advanced stage, available therapies are restricted to palliative care and local treatment and have no satisfactory effect [5]. The current methods of HCC diagnosis are divided into two main categories: imaging and biomarker tests. Cancer biomarkers are measurable molecules or substances observed in cells, tissues or body fluids, and their level predicts the presence of cancer or even indicates the stage of cancer. The measurement of biomarkers always leads to a specific numeric value, and the obtained result, although not necessarily accurate, is generally more objective than images produced by imaging techniques, since their interpretation is often subjected to the judgment and experience of physicians. Additionally, there is a strong economic argument for the acceptance of biomarkers in cancer detection, especially in the countries where advanced imaging instruments are limited or even unavailable [5–8].

Recent studies have identified a number of biomarkers in the early detection of HCC, including alpha-fetoprotein (AFP) [9–11], lens culinaris agglutinin-reactive alpha-fetoprotein (AFP-L3) [10,12–13], des-gamma-carboxyprothrombin (DCP) [9–10,13], glypican-3 (GPC-3) [14–15], cytokeratin 19 (CK19) [15], golgi protein 73 (GP73) [16], microRNA (miRNA) [17–18], osteopontin (OPN) [11,19], annexin A2 [20] and midkine (MDK) [21]. According to the five-phase program adopted by the Early Detection Research Network (EDRN) of the National Cancer Institute (NCI), most HCC biomarkers have been evaluated in phase II and III studies, and further investigations are needed to determine their utility for clinical purposes. AFP is the only HCC biomarker that has been studied through all five phases of biomarker development. This biomarker has been the most commonly used one as an early screening and diagnosis tool of HCC [7–8]. The average level of serum AFP in the healthy person is less than 20 ng/mL [22], but high levels, sometimes exceeding 100 µg/mL, can be found in the serum of patients suffering from HCC [23].

**Figure 1:**
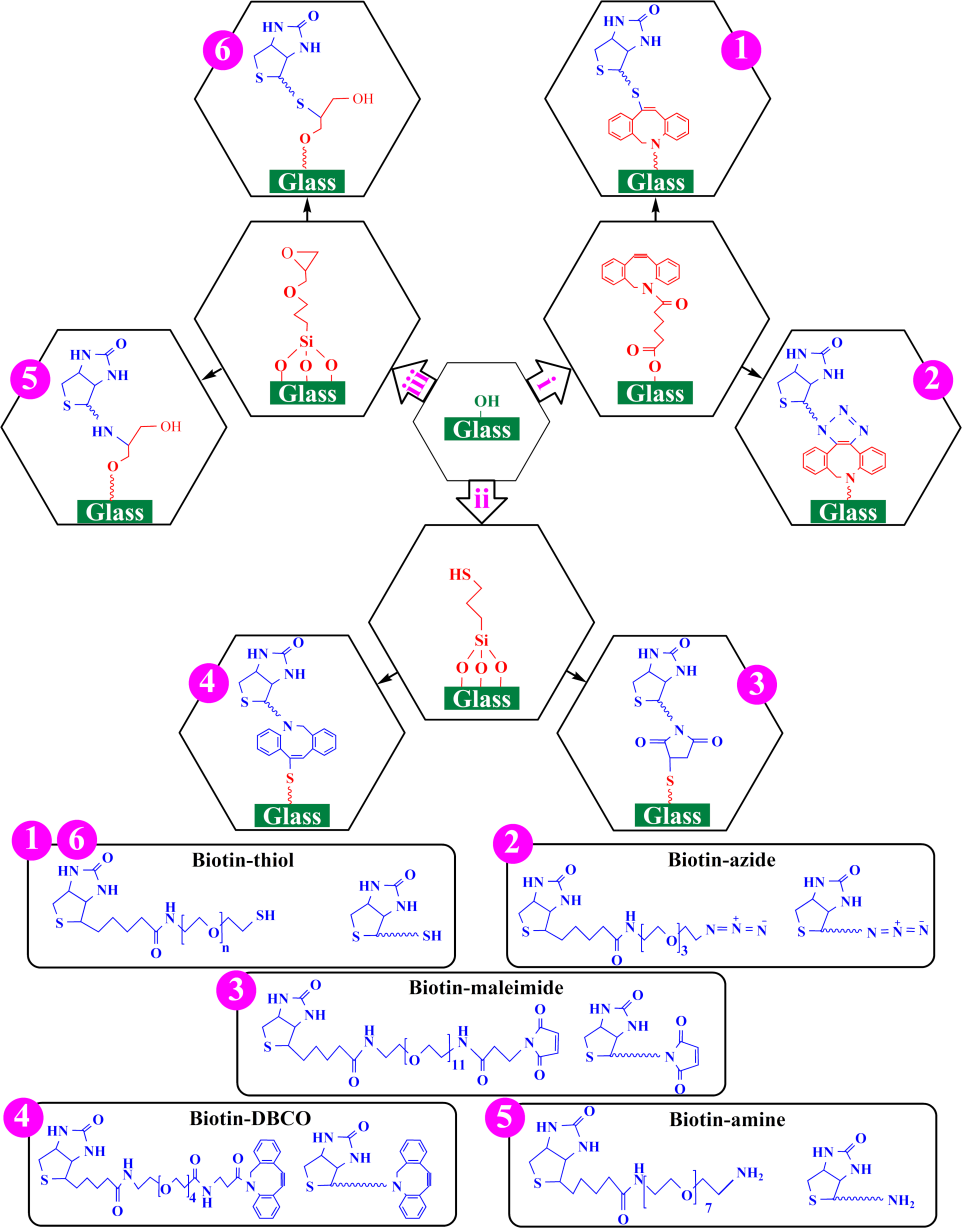
Biotin immobilization on the hydroxy-terminated glasses via different click reactions. Hydroxy-terminated glasses were first functionalized with DBCO–OH (path i), (3-mercaptopropyl)trimethoxysilane (MPTMS) (path ii) or (3-glycidyloxypropyl)trimethoxysilane (GPTMS) (path iii) and subsequently biotinylated through different click reactions (routes 1–6).

In this study, we compare different approaches of binding chemistry for the construction of sensitive fluorescent immunosensors for AFP detection by combining the unique characteristics of click chemistry with the high sensitivity of the biotin–streptavidin–biotin sandwich strategy as well as the high selectivity of antigen–antibody interactions. Six different fabrication routes based on dibenzocyclooctyne- (DBCO-), thiol- and epoxy-functionalization of glasses and click chemistry attachment of biotinylated molecules (Figure 1) were used to obtain biotinylated surfaces for the construction of the AFP detection sandwich. Finally, the best performing fabrication route was evaluated for its efficiency as sensor platform. Click chemistry reactions, which have been widely studied since their introduction by Kolb, Finn and Sharpless in 2001 [24], have unique advantages such as high reaction rates, high yield, mild reaction conditions and easy post-treatment [25–29]. Afterwards, the biotinylated glasses produced following the six different functionalization procedures, which are referred to as routes 1–6 throughout this article, were gradually incubated with solutions of streptavidin and biotinylated anti-AFP to obtain a biotin–streptavidin–biotin sandwich structure. The biotin–streptavidin–biotin sandwich strategy is used in the fabrication of immunosensors due to the improved sensitivity [30–32]. Lastly, fluorescent micropatterns were fabricated by microchannel cantilever spotting (μCS). Here, patterns are written with fluorescently labeled AFP (as antigen) on the surfaces modified with anti-AFP (as antibody). In different studies, many selective biosensors have been developed based on antigen–antibody interactions [22,30–31,33–37]. The technique used in this work provides a new platform for the fabrication of clinical immunosensors, which is applicable for the fast and effective diagnosis of HCC.

## Results and Discussion

### Generation of biotinylated surfaces

To generate biotinylated surfaces as a basis for the sensor antibody sandwich, six different fabrication routes were followed (Figure 1). First, the glass substrates were plasma-treated to generate reactive hydroxy groups on their surfaces and then functionalized with either acid (to obtain DBCO-terminated surfaces) or silanes (to obtain thiol- and epoxy-terminated surfaces). After base functionalization, biotin was immobilized on the DBCO-, thiol- or epoxy-functionalized glasses by incubation with the respective click chemistry partner molecules leading to the six different biotinylation routes: for DBCO-terminated glass, biotin–thiol (route 1) and biotin–azide (route 2) were used, for thiol-terminated glass, biotin–maleimide (route 3) and biotin–DBCO (route 4) were used and for epoxy-terminated glass, biotin–amine (route 5) and biotin–thiol (route 6) were employed. The six different surfaces were subsequently characterized, which is mandatory for their successful implementation and a thorough comparison of their properties.

### Characterization of the surfaces by XPS and AFM

**Figure 2:**
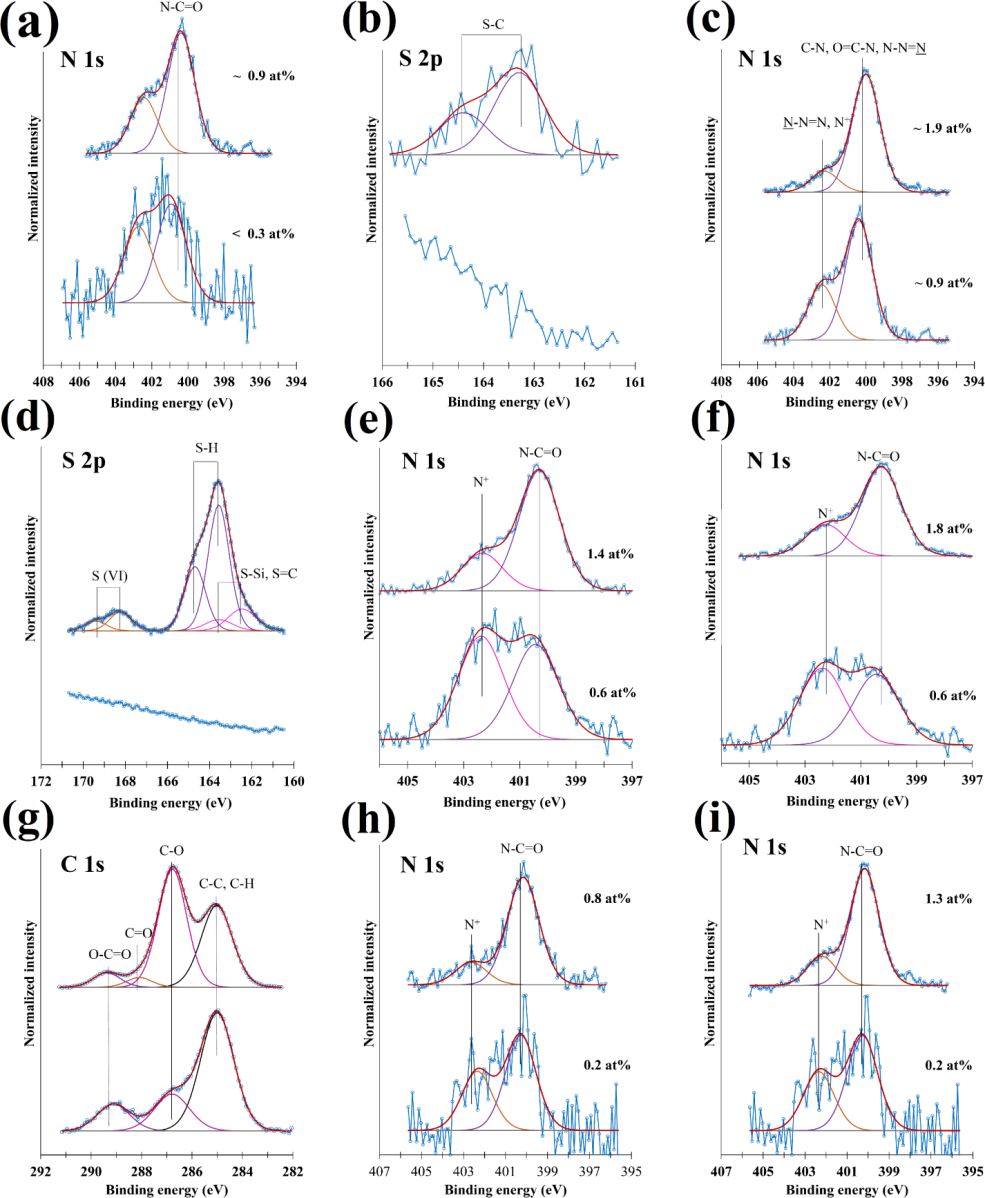
XPS characterization of a) the hydroxy-terminated (bottom) and the DBCO-terminated (top) glasses, b) the DBCO-terminated glass (bottom) and the sample of route 1 (top), c) the DBCO-terminated glass (bottom) and the sample of route 2 (top), d) the hydroxy-terminated (bottom) and the thiol-terminated (top) glasses, e) the thiol-terminated glass (bottom) and the sample of route 3 (top), f) the thiol-terminated glass (bottom) and the sample of route 4 (top), g) the hydroxy-terminated (bottom) and the epoxy-terminated (top) glasses, h) the epoxy-terminated glass (bottom) and the sample of route 5 (top), i) the epoxy-terminated glass (bottom) and the sample of route 6 (top).

All steps of the immobilization reactions were monitored by X-ray photoelectron spectroscopy (XPS) to validate the expected chemical reactions taking place (Figure 2). The functionalization of the glasses with DBCO–OH is proven by the appearance of a peak at 400.4 eV attributed to the N–C=O groups present in DBCO [38] (Figure 2a). For the sample produced following route 2, the nitrogen concentration increases clearly after the reaction with the biotin–azide, and the absence of a peak around 405 eV [39–40] proves the reaction of the azide to a triazole ring (Figure 2c). For the sample produced via route 1, the attachment of the biotin–thiol is shown here by the presence of the S 2p doublet with S 2p_3/2_ at 163.3 eV [41] (Figure 2b).

Similarly, the thiol-terminated glass presents a clear sulfur signal attributed to the thiol and a weak component at 168.0 eV probably due to the oxidation of some sulfur atoms (Figure 2d). The next steps (route 3 and 4) can be followed by the increase of the nitrogen content at the surface (Figure 2e and Figure 2f). The C 1s peak (Figure 2g) indicates the functionalization with (3-glycidyloxypropyl)trimethoxysilane (GPTMS) especially given the pronounced increase of the intensity of the component at 286.6 eV [42] attributed to the C–O moiety. For the samples of routes 5 and 6 (Figure 2h and Figure 2i), the clear signals attributed to nitrogen occurring in the spectra at 400.0 eV also result from the successful reactions. To confirm the quality of the functionalized layers, after each step of the functionalization process, the roughness of the samples was monitored by atomic force microscopy (AFM). The results are shown in Supporting Information File 1, Figure S1. While the roughness increases slightly over the course of functionalization, overall, the samples remain relatively smooth with root-mean-square roughness values (*R*_q_) below 1 nm, showing a homogeneous reaction built-up without introduction of a nanotexture that might lead to confounding factors in the evaluation of surface binding.

### Comparison of the immobilization routes for the sandwich application

**Figure 3:**
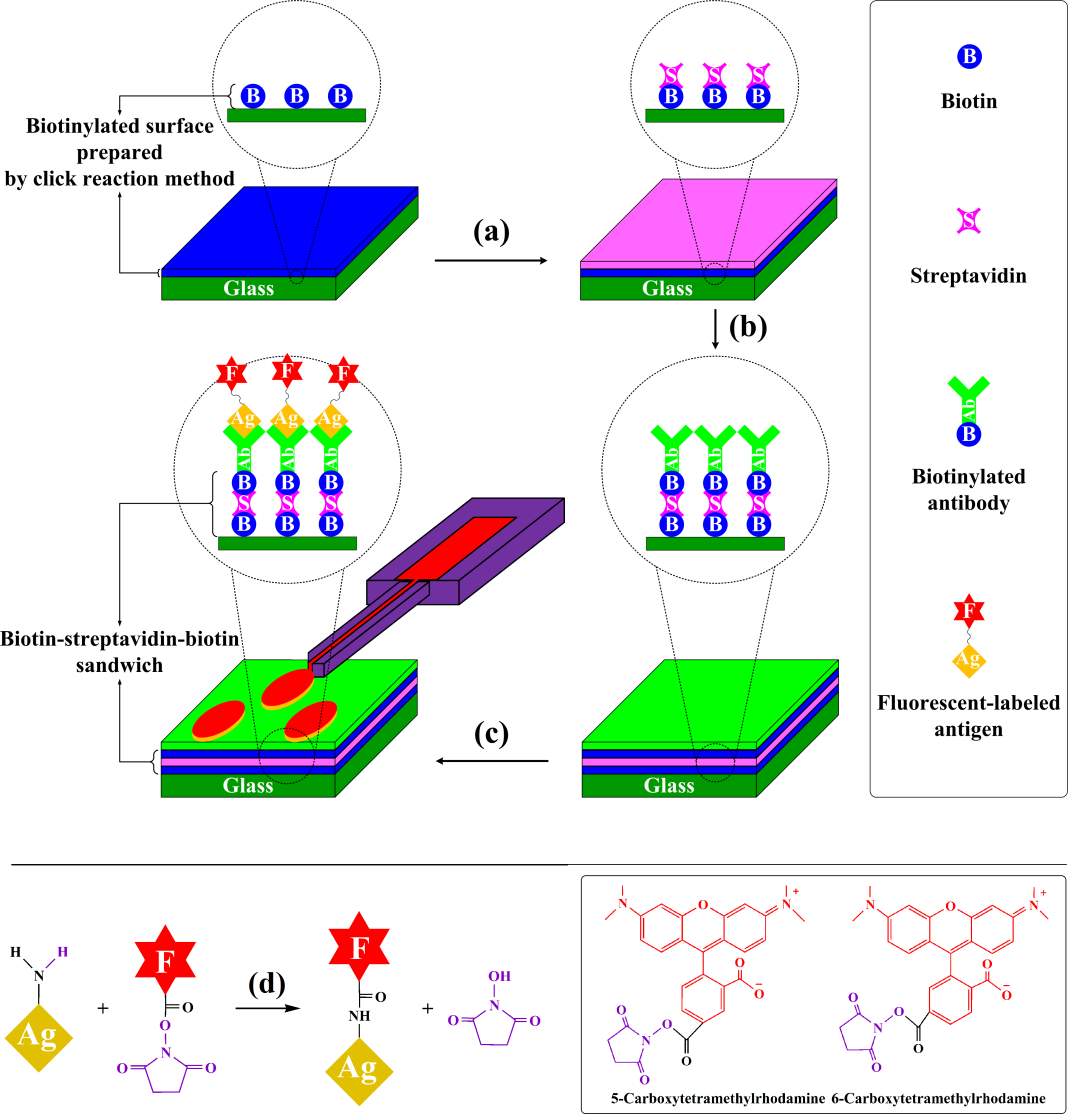
Schematic of the microarrays prepared for AFP detection. a) Incubating biotinylated surfaces with streptavidin solution, b) reaction of the streptavidin on the surface with the biotinylated anti-AFP, c) delivery of fluorescently labeled AFP via µCS, and d) preparation of fluorescently labeled AFP through the reaction between the NHS-ester reagent (NHS–rhodamine) and the primary amine of the antigen (AFP).

The performance of the different types of biotinylated surfaces was probed by addition of a biotin–streptavidin–biotin sandwich structure for antibody immobilization (Figure 3). To this end, the surfaces were first incubated with streptavidin and subsequently with a biotinylated anti-AFP antibody leading to a surface homogeneously covered by the antigen binding antibody. To evaluate the amount of antigen that the different surfaces can bind, fluorescently labeled AFP was then spotted onto the surfaces via µCS.

**Figure 4:**
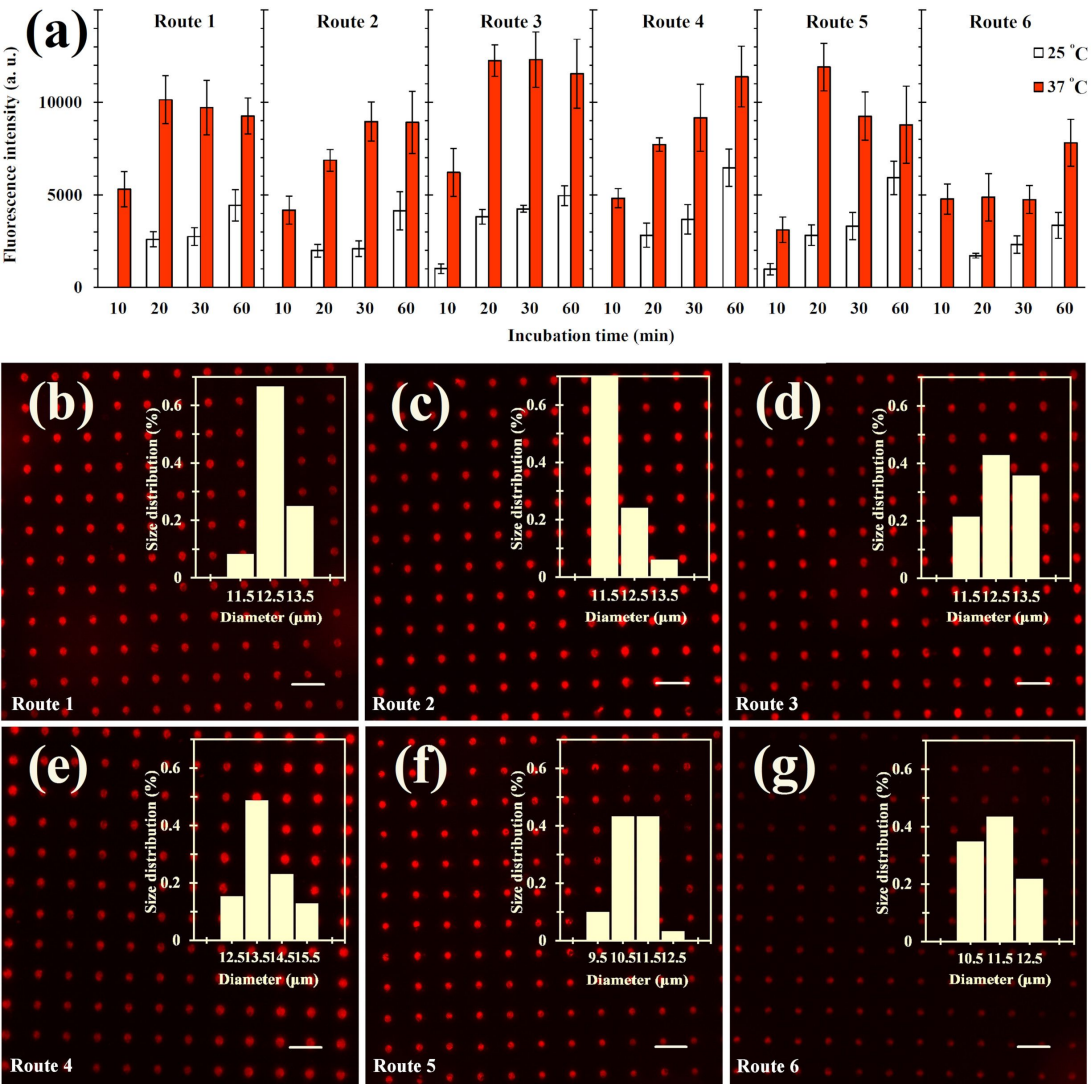
Detection of the AFP antigen. a) Fluorescence of the microarrays after incubating fluorescently labeled AFP spots with anti-AFP on the surfaces at different times (10, 20, 30 and 60 min) and at two temperatures (25 and 37 °C). Anti-AFP was immobilized on the different biotinylated surfaces using the biotin–streptavidin–biotin sandwich technique previously prepared via different click reaction methods (samples of routes 1–6). b–g) Fluorescence microscope images of the micropatterns obtained at the optimum incubation time and temperature. All microarrays were spotted at a relative humidity of 20%. The ink concentration was 800 μg/mL. Dwell and exposure times of all images were 0.1 and 0.4 s, respectively. The corresponding spot size distribution is given in the insets. All scale bars are equal to 50 μm.

The microarrays were incubated for performing antigen–antibody interaction for different times (10 to 60 min) and at different temperatures (room temperature of 25 °C and elevated physiological temperature of 37 °C). Then, the samples were washed and the fluorescence signal of the spot array was quantified (Figure 4). As a general trend, higher fluorescence intensities are observed at an elevated temperature of 37 °C for all microarrays at the same incubation time. For room temperature incubation, the observed fluorescence intensity increases with incubation time for all routes within the monitored time frame of 10 to 60 min. In the case of incubation at 37 °C, some routes exhibit the same behavior (routes 2, 4, and 6) while the others (routes 1, 3 and 5) exhibit an intensity peak after some time before the fluorescence intensity decreases again. This behavior is probably caused by the drying effect that becomes more pronounced at elevated temperatures since the solvent evaporation is enhanced. When the material is dried out (and is thus only physisorbed onto the surface), it is more easily removed in the washing step before the fluorescence measurement. Based on the results of the fluorescence analysis, an incubation of samples of either route 3 or 5 at 37 °C for 20 min was determined to offer an optimal performance.

### Evaluation of sensitivity

**Figure 5:**
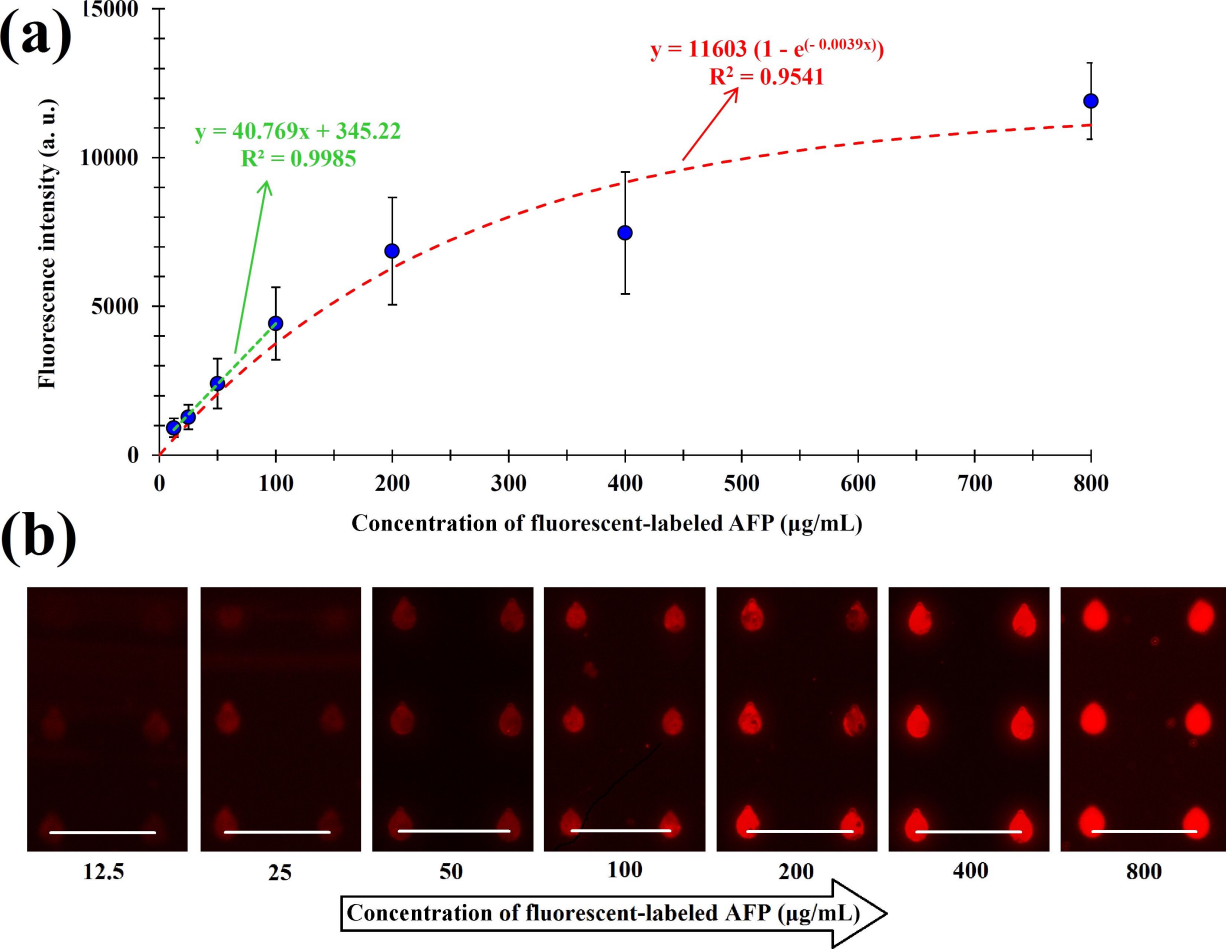
Evaluation of the sensitivity of the microarrays prepared from the sample of route 5 after incubating anti-AFP spots with fluorescently labeled AFP. a) Fluorescence intensity of the microarrays at different concentrations of fluorescently labeled AFP (12.5, 25, 50, 100, 200, 400 and 800 μg/mL). b) Fluorescence microscope images of the micropatterns. All microarrays were spotted at a relative humidity of 20%. The dwell and exposure times of all images were 0.1 and 0.4 s, respectively. As incubation conditions we chose 37 °C and 20 min. All scale bars are equal to 50 μm.

To further elucidate the capability of the approach for sensing applications, a sensitivity curve for one of the optimal samples (route 5, 37 °C, 20 min) was measured. Here, the optimal microarray was incubated with different concentrations of the fluorescently labeled AFP (ranging from 12.5 to 800 µg/mL). The resulting fluorescence intensity curve and corresponding representative images are presented in Figure 5.

When extrapolating the low-concentration part in the linear regime of the curve (y = 40.77*x* + 345.22) and taking into account the fluorescence background in the different experiments (745.58 ± 118.32 a.u.), a sensitivity limit of the detection of 9.8 ± 2.9 µg/mL is obtained for the given conditions of our setup. Negative control samples (no AFP present) yielded no fluorescence signal (Supporting Information File 1, Figure S2a). Furthermore, unspecific binding of non-target proteins is assumed to be low as revealed by a control experiment with fluorescently labeled streptavidin as model protein. Here, the fluorescence remaining after washing of the microarray is highly reduced (Figure S2b). Finally, it should be noted that unlabeled AFP can also be detected in this approach. For demonstration, unlabeled AFP was spotted by µCS and stained via the same type of biotinylated antibodies as immobilized on the surface. This enables the identification of the sites were unlabeled AFP bound by subsequent staining with fluorescently labeled streptavidin (Figure S3). After incubation a fluorescent microarray pattern becomes visible again (Figure S4).

## Conclusion

In this study, we present the implementation of a sensitive fluorescent immunosensor for the detection of AFP, which is used as a common cancer-related model protein. We compared the AFP microarray sensors resulting from six different fabrication routes based on different functionalization methods (DBCO-, thiol- and epoxy-termination) and subsequent click chemistry immobilization of biotin. In the present setup, the functionalization by thiol–silane with subsequent biotin immobilization by biotin–maleimide as well as the functionalization by epoxy–silane with subsequent biotin immobilization by biotin–amine yielded the best performance of the corresponding microarray sensors. The sensitivity of the epoxy–amine-based array was evaluated to be 9.8 ± 2.9 µg/mL, providing a rapid and inexpensive screening sensor compared to the more sensitive, but also much more elaborate detection approaches. Moreover, the approach can be extended towards label-free detection. To this end, a sandwich strategy is employed by attaching a second biotinylated antibody and a fluorescently labeled streptavidin. Further sensitivity improvements are expected upon utilizing novel antifouling and special wettability surfaces [43–44]. Our results highlight the utility of binding chemistry in the building of highly sensitive protein detection sensors needed, for example, in cancer biomarker detection.

## Experimental

### Chemicals

Table 1 lists the most important materials used in this study. All other materials were of analytical grade and were used as-received without extra purification steps.

**Table 1:** Overview of the materials used in the experiments.

Commercial name	Short name	Role	Source

biotin PEG thiol, MW 2000	biotin–thiol	biotinylated molecule	Nanocs Company (USA)
azide–PEG_3_–biotin conjugate	biotin–azide	biotinylated molecule	Jena Bioscience (Germany)
biotin–dPEG^®^_11_-MAL	biotin–maleimide	biotinylated molecule	Sigma-Aldrich (Germany)
dibenzylcyclooctyne–PEG_4_–biotin conjugate	biotin–DBCO	biotinylated molecule	Jena Bioscience (Germany)
biotin–dPEG^®^_7_–NH_2_	biotin–amine	biotinylated molecule	Sigma-Aldrich (Germany)
dibenzocyclooctyne-acid	DBCO-acid	coupling agent in esterification	Jena Bioscience (Germany)
(3-mercaptopropyl)trimethoxysilane	MPTMS	coupling agent in silanization	Sigma-Aldrich (Germany)
(3-glycidyloxypropyl)trimethoxysilane	GPTMS	coupling agent in silanization	Sigma-Aldrich (Germany)
*N*,*N'*-dicyclohexylcarbodiimide	DCC	catalyst	Sigma-Aldrich (Germany)
4-dimethylaminopyridine	DMAP	catalyst	Sigma-Aldrich (Germany)
triethylamine	TEA	catalyst	Sigma-Aldrich (Germany)
bismuth(III) trifluoromethanesulfonate	Bi(OTf)_3_	catalyst	Sigma-Aldrich (Germany)
streptavidin	S	conjugation with biotinylated molecules	Sigma-Aldrich (Germany)
streptavidin–Cy3 (fluorescently labeled streptavidin)	F–S	conjugation with biotinylated molecules	Sigma-Aldrich (Germany)
alpha-fetoprotein (source: human cord serum)	AFP or Ag	biomarker or antigen	Lee BioSolutions, Inc. (USA)
AFP antibody (C3) [biotin]	B-Ab	biotinylated antibody or biotinylated anti-AFP	Novus Biologicals (USA)
5-(and 6)-carboxytetramethylrhodamine, succinimidyl ester	NHS-rhodamine	fluorescent reagent for labeling	Thermo Scientific (USA)
phosphate buffered saline	PBS	buffer	Sigma-Aldrich (Germany)
dimethyl sulfoxide	DMSO	solvent	Sigma-Aldrich (Germany)

### Preparation of biotinylated substrates

Standard glass coverslips (1 × 1 cm, VWR, Germany) were cleaned with chloroform, 2-propanol and deionized water, dried by blowing with nitrogen and exposed to oxygen plasma (10 sccm O_2_, 0.2 mbar and 100 W) for 2 min. The obtained hydroxy-terminated glasses were first functionalized with DBCO-acid, MPTMS or GPTMS and subsequently biotinylated via different click reactions as follows:

#### Functionalization

To obtain DBCO-functionalized surfaces, the hydroxy-terminated glasses were immersed in solutions of DBCO-acid, DCC and DMAP in DMSO for 24 h at room temperature (Figure 1, path i). Thiol-functionalized surfaces were obtained by soaking the hydroxy-terminated glasses in a 2% v/v solution of MPTMS in toluene for 5 h at room temperature (Figure 1, path ii). To obtain epoxy-functionalized surfaces, the hydroxy-terminated glasses were immersed in a 2% v/v solution of GPTMS in toluene for 5 h at room temperature (Figure 1, path iii). All the functionalized glasses were washed sequentially with deionized water and suitable solvents to remove unreacted materials and dried under a nitrogen stream.

#### Biotin immobilization

Different click reactions were used for the immobilization of biotin on the DBCO-, thiol- or epoxy-functionalized surfaces (Figure 1, routes 1–6). For all reactions routes, biotin solutions of the same concentration (2 μmol/mL in DMSO) were selected. The reactions proceeded within 20 min at 37 °C. The samples of routes 1 and 2 were prepared by immersing the DBCO-functionalized glasses in solutions of biotin–thiol (*M*_w_ = 2000 g/mol, 4000 μg/mL) and biotin–azide (*M*_w_ = 445 g/mol and 890 μg/mL), respectively. For routes 3 and 4, the samples were provided by soaking the thiol-functionalized glasses in solutions of biotin–maleimide (*M*_w_ = 922 g/mol, 1844 μg/mL) and biotin–DBCO (*M*_w_ = 750 g/mol, 1500 μg/mL), respectively. The reaction between thiol and maleimide was catalyzed by 10 mol % triethylamine (TEA) to biotin–maleimide added to the solution. The samples of routes 5 and 6 were obtained by adding epoxy-functionalized glasses to solutions of biotin–amine (*M*_w_ = 595 g/mol, 1190 μg/mL) and biotin–thiol (*M*_w_ = 2000 g/mol, 4000 μg/mL), respectively. For the reaction between epoxy and amine, 1 mol % Bi(OTf)_3_ to biotin–amine was added as a catalyst to the biotin–amine solution. The reaction between epoxy and thiol was catalyzed by 10 mol % TEA to biotin–thiol. After incubation, the excess ink solution was rinsed with deionized water and suitable solvents and the samples were dried in a nitrogen stream.

#### Antibody attachment via sandwich

To detect the AFP antigen, anti-AFP was coated on the glass as antibody (Ab). As shown in Figure 2a and Figure 2b, the antibody coating was performed in two steps. First, the biotinylated surfaces were incubated for 1 h in a solution of 10 μg/mL of streptavidin in phosphate buffered saline (PBS). The samples were washed with PBS and deionized water and were blow-dried with nitrogen. Second, a solution of 10 μg/mL of the biotinylated antibody (B-Ab) was made to react with the streptavidin molecules on the surface. The resulting antibody-terminated surfaces were rinsed with PBS to remove any excess solution and were dried with nitrogen.

#### Microarray writing via µCS

To study the interaction between antibody and antigen, fluorescently labeled AFP ink was spotted onto the antibody-terminated surfaces by µCS within 15 × 15 spot arrays. The spotting process was performed on a nanolithography platform (NLP 2000 system, NanoInk, USA) utilizing surface patterning tool cantilevers (SPT-S-C10S, Bioforce Nanosciences) [45–46]. Prior to use, the cantilevers were activated by oxygen plasma (10 sccm O_2_, 0.2 mbar, 100 W, 2 min) and used immediately. The pen reservoir was filled with 0.2 μL of ink solution. For all patterns, a probe dwell time of 0.1 s was applied. Spotting was performed at an optimized relative humidity of 20%, which was determined in the previous study [25]. Figure 2c illustrates a schematic picture of the lithography process. To study the influence of time and temperature on the coupling content, after lithography, the reaction between fluorescently labeled AFP and anti-AFP was allowed to proceed for 10, 20, 30 and 60 min at either 25 or 37 °C in a temperature chamber (PU-1KP, ESEPC, Japan). After incubation, the excess ink solution was rinsed with deionized water and the surface was dried in a nitrogen stream.

#### Preparation of fluorescently labeled AFP ink

Succinimidyl-ester labeling reagents are the simplest and most frequently used reagents for labeling proteins like antibodies. Within a pH range of 7–9, succinimidyl-ester reacts efficiently with the primary amino groups (–NH_2_) in the side chain of the lysine (K) residues of proteins to form stable amide bonds. This reaction results in the release of *N*-hydroxysuccinimide (NHS). For labeling AFP, we used NHS–rhodamine, a succinimidyl-ester functional group attached to the rhodamine core. This fluorescent labeling reagent absorbs green visible light (552 nm) and emits orange-red visible light (575 nm). Before labeling, the AFP solution was dialyzed in PBS to replace the tris(hydroxymethyl)aminomethane buffer solution with PBS. For AFP labeling, NHS–rhodamine was added to the AFP solution at a 10- to 15-fold molar excess and dialyzed again to remove any extra NHS–rhodamine (Figure 2d).

### Surface characterization

The surface of the bare and functionalized glasses was characterized using surface-sensitive techniques, including atomic force microscopy (AFM) and X-ray photoelectron spectroscopy (XPS). To map the surface roughness, AFM in tapping mode was conducted with a Dimension Icon (Bruker, Germany) device with HQ:NSC15/Al BS cantilevers (MikroMasch, USA). All measurements were done in air and under ambient conditions. As a measure of the roughness, the root-mean-square average of the height deviations with regard to the mean image data plane (*R*_q_ in the software) was sampled from 5 × 5 μm^2^ images (three per sample) using the AFM system onboard software (NanoScope 8.10, Bruker, Germany). The XPS analysis was performed using a K-Alpha+ XPS spectrometer (ThermoFisher Scientific, East Grinstead, UK) using the Thermo Avantage software as previously described [47]. Sample analysis was performed as reported in [25].

### Fluorescence imaging

The fluorescently labeled surface patterns were imaged using a Nikon Eclipse 80i upright fluorescence microscope (Nikon, Japan) equipped with an Intensilight illumination (Nikon, Japan), a CoolSNAP HQ2 camera (Photometrics, USA) and a Texas Red set (Y-2E/C, Nikon).

### Statistical analysis

The data shown in Figure 4 and Figure 5 were expressed as mean ± standard deviation. Significant differences of the arrays resulting from the different treatment routes were analyzed by one-way analysis of the variance (ANOVA) and the Duncan tests at *p* < 0.05 using the statistical package for the social sciences (SPSS) software version 19.0.0 (Abacus Concepts Inc., Berkeley, California, USA).

## Supporting Information

File 1 Additional figures.
