# Supplementary material for: Evaluation of click chemistry microarrays for immunosensing of alpha-fetoprotein (AFP)
Source: Beilstein J Nanotechnol. 2019 Dec 16;10:2505–15. doi: 10.3762/bjnano.10.241 (PMC6941445; doi:10.3762/bjnano.10.241)
Supplement: File 1 — Additional figures. [file Beilstein_J_Nanotechnol-10-2505-s001.pdf]

BEILSTEIN JOURNAL OF NANOTECHNOLOGY

Supporting Information

for

Evaluation of click chemistry microarrays for immunosensing of alpha-fetoprotein (AFP)

Seyed Mohammad Mahdi Dadfar, Sylwia Sekula-Neuner, Vanessa Trouillet, Hui-Yu Liu, Ravi Kumar, Annie K. Powell and Michael Hirtz

Beilstein J. Nanotechnol. **2019,** *10,* 2505–2515. doi:10.3762/bjnano.10.241

Additional figures

License and Terms: This is a supporting information file under the terms of the Creative Commons Attribution License (https://creativecommons.org/licenses/by/4.0). Please note that the reuse, redistribution and reproduction in particular requires that the authors and source are credited.
The license is subject to the *Beilstein Journal of Nanotechnology* terms and conditions: (https://www.beilstein-journals.org/bjnano)

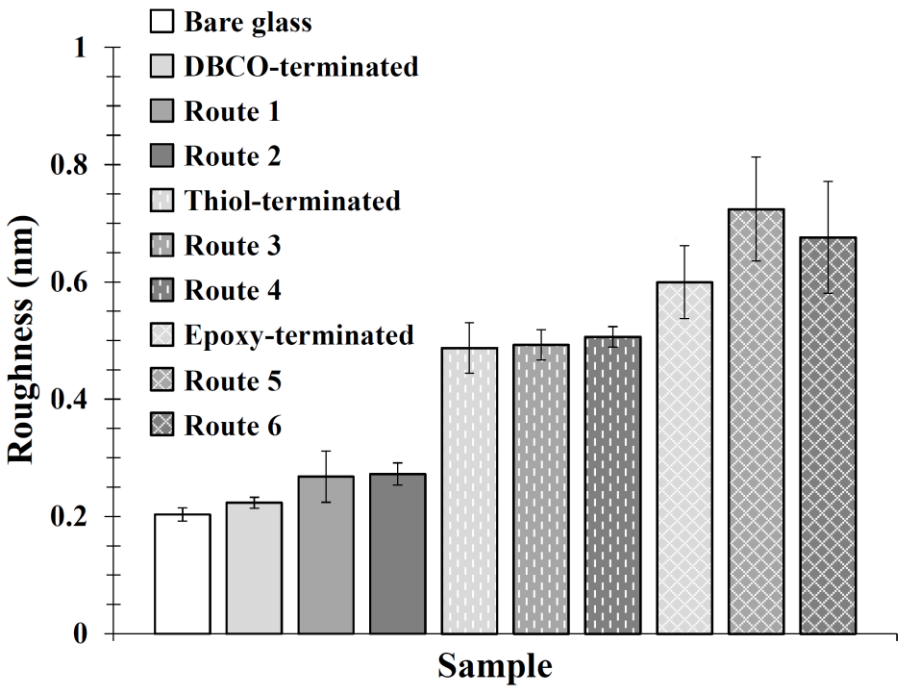

Figure S1: Roughness values determined by AFM test for the bare glass and hydroxyl-, DBCO-, thiol- and epoxy-terminated glasses as well as for samples of routes 1–6. The bare glass features a roughness of 0.20 ± 0.01 nm. While DBCO functionalization by acid only slightly increases the roughness to 0.22 ± 0.01 nm, the silanization of the thiol- and epoxy-terminated glasses leads to significantly higher roughness (0.49 ± 0.04 nm and 0.60 ± 0.06 nm, respectively). This difference in roughness is probably caused by the possibility of crosslinking between silanes leading to a rougher surface. The next step in the different routes, the immobilization of biotin via a matching click reaction, does increase the roughness only slightly or even not significantly at all: on the DBCO-functionalized surfaces, adding of biotin-thiol (route 1) leads to a slightly increased roughness of 0.27 ± 0.04 nm, and biotin-azide (route 2) yields 0.27 ± 0.02 nm. On the thiol-terminated glass, no significant further increase of the roughness is observed for biotin-maleimide (route 3) with 0.50 ± 0.03 nm and biotin-DBCO (route 4) with 0.51 ± 0.02 nm. The epoxy-terminated glass shows a slight increase in roughness for biotin-amine (route 5) with 0.72 ± 0.09 nm and biotin-thiol (route 6) with 0.68 ± 0.10 nm.

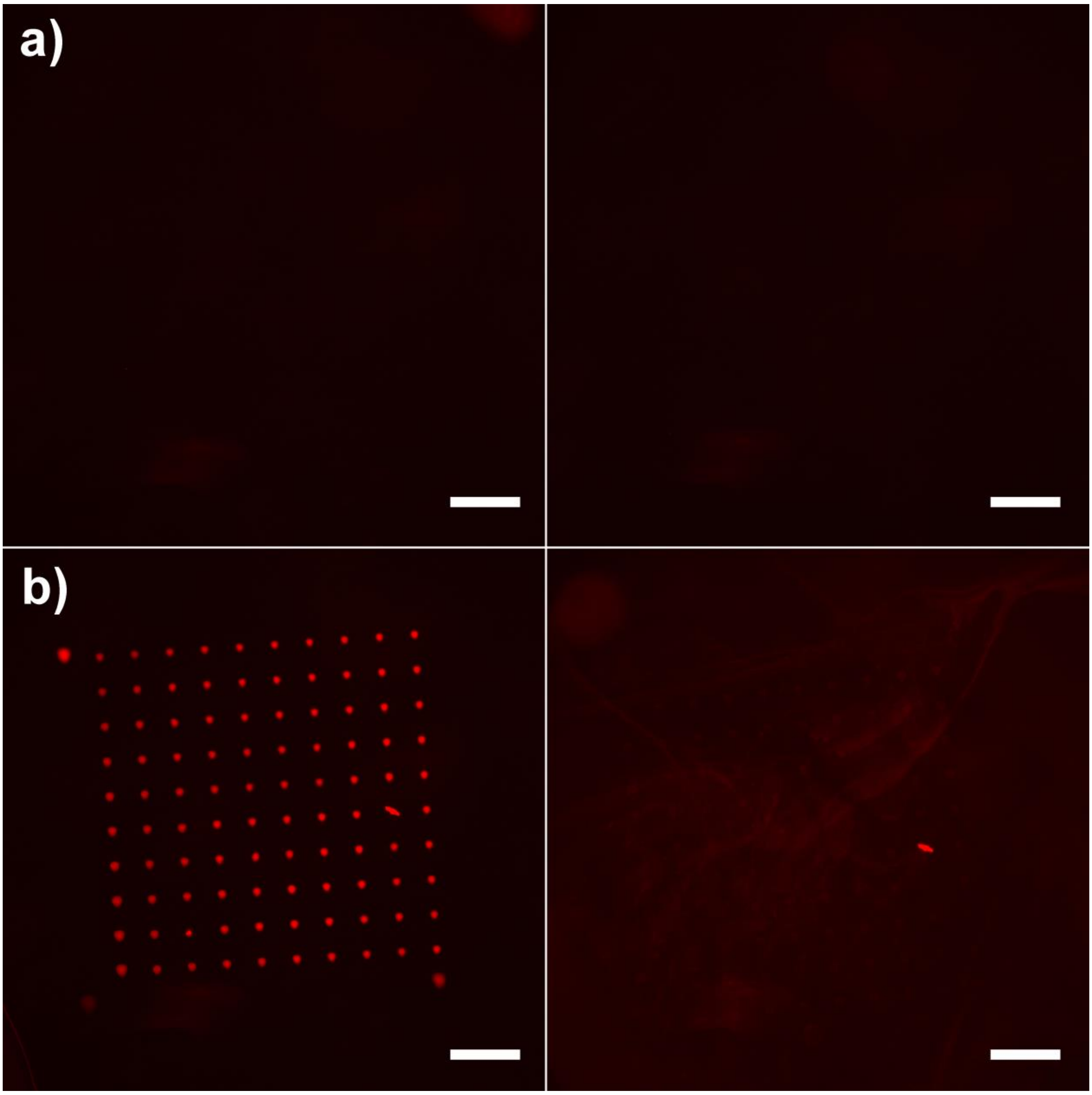

Figure S2: Control experiments for AFP sensing. a) negative control (no AFP present) before (left) and after (right) washing. No fluorescence is detected. b) control with Cy3 labeled streptavidin (50 µg/mL) before (left) and after (right) washing. The fluorescence intensity before and after washing decreases from (9622.3 ± 655.8) a.u. to (384.6 ± 180.4) a.u. indicating only minor unspecific binding of the labeled streptavidin. Scale bars equal 100 µm in all images.

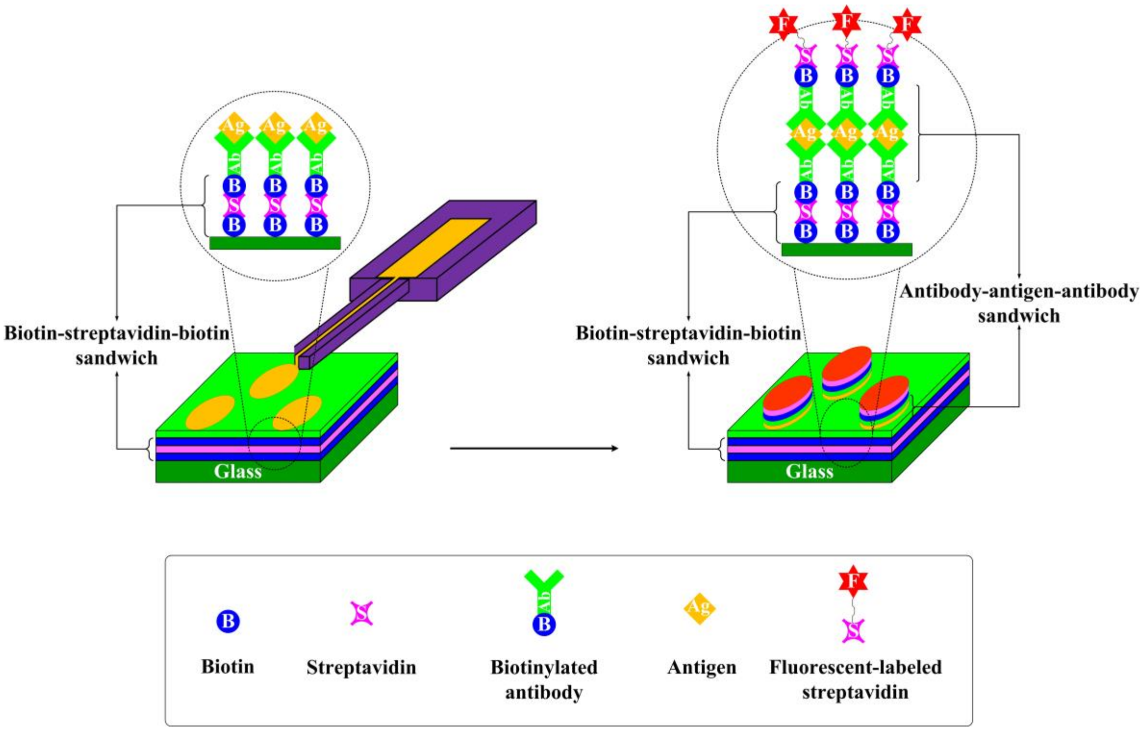

Figure S3: Detection of unlabeled AFP by the additional sandwich approach via a second binding of the biotinylated antibody and fluorescent-labeled streptavidin.

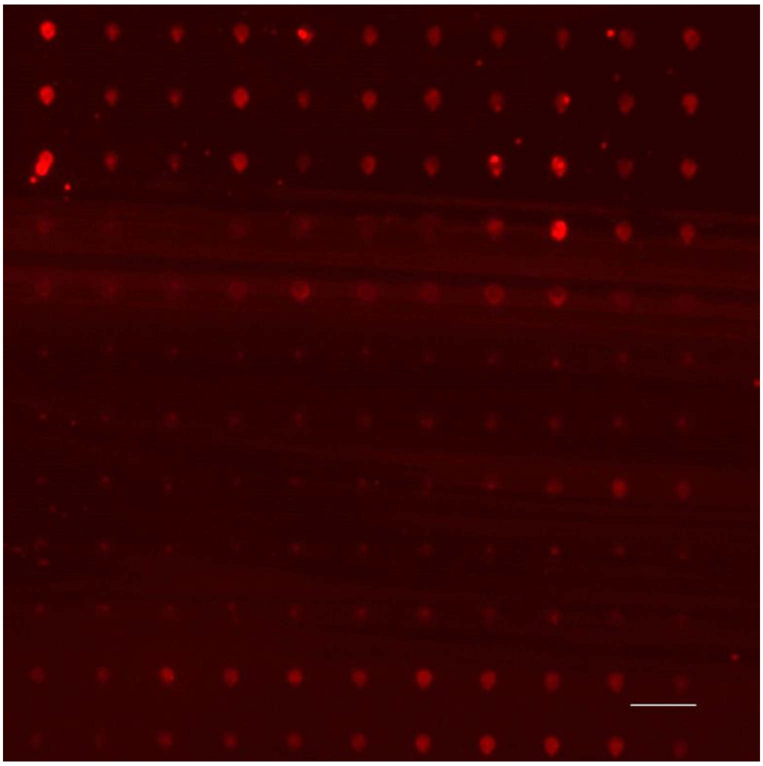

Figure S4: Fluorescence microscope image of the micropattern obtained from unlabeled AFP after implementation of the incubation step with fluorescent-labeled streptavidin. Scale bar equals 50 µm.
